# Child-Na Score: A Predictive Model for Survival in Cirrhotic Patients with Symptomatic Portal Hypertension Treated with TIPS

**DOI:** 10.1371/journal.pone.0079637

**Published:** 2013-11-11

**Authors:** Hui Chen, Ming Bai, Xingshun Qi, Lei Liu, Chuangye He, Zhanxin Yin, Daiming Fan, Guohong Han

**Affiliations:** 1 Department of Liver Disease and Digestive Interventional Radiology, Xijing Hospital of Digestive Diseases, Fourth Military Medical University, Xi’an, China; 2 State Key Laboratory of Cancer Biology and Xijing Hospital of Digestive Diseases, Fourth Military Medical University, Xi’an, China; Northwestern University Feinberg School of Medicine, United States of America

## Abstract

**Background and Aim:**

Several models have been developed to predict survival in patients with cirrhosis undergoing TIPS; however, few of these models have gained widespread acceptance, especially in the era of covered stents. The aim of this study was to establish an evidence-based model for predicting survival after TIPS procedures.

**Methods:**

A total of 210 patients with cirrhosis treated with TIPS were considered in the study. We comprehensively investigated factors associated with one-year survival and developed a new predictive model using the Cox regression model.

**Results:**

In the multivariate analysis, the Child-Pugh score and serum sodium levels were independent predictors of one-year survival. A new score incorporating serum sodium into the Child-Pugh score was developed: Child-Na score. We compared the predictive accuracy of Child-Na score with that of other scores; only the Child-Na and MELD-Na scores had adequate predictive ability in patients with serum Na levels <138 mmol/L. The best Child-Na cut-off score (15.5) differentiated two groups of patients with distinct prognoses (one-year cumulative survival rates of 80.6% and 45.5%); this finding was confirmed in a validation cohort (n = 86). In a subgroup analysis stratifying patients by indication for TIPS, the Child-Na score distinguished patients with different prognoses.

**Conclusions:**

Patients with variceal bleeding and a Child-Na score ≤15 had a better prognosis than patients with a score ≥16. Patients with refractory ascites and a Child-Na score ≥16 had a high risk of death after the TIPS procedures; caution should be used when treating these patients with TIPS.

## Introduction

The transjugular intrahepatic portosystemic shunt (TIPS) procedure is increasingly used to treat the complications of portal hypertension. TIPS evolved from a life-saving procedure to a procedure used to improve a patient's survival and quality of life [1]. The major drawback of TIPS is the potential occurrence of shunt dysfunction and hepatic encephalopathy (HE). The use of expanded polytetrafluoroethylene (ePTFE)-covered stent-grafts has overcome the problem of post-TIPS shunt insufficiency [2], and the 2009 update of the American Association for the Study of Liver Diseases (AASLD) Practice Guidelines states that “the use of ePTFE-covered stents is now preferred” [3].

The one-year mortality rate following TIPS with the use of covered stents is partially dependent on the indication for the procedure, and mortality has been found to range from 10-35% [4]-[11]. Ideally, if a prognostic score could accurately predict the survival of patients after TIPS placement, this score could identify patients with an expected survival benefit and patients whose condition is likely to deteriorate; the patients with a poor expected prognosis could then be moved up on the liver transplantation list.

Several models have been developed in an attempt to identify reliable predictors of the short- and long-term mortality rates of patients undergoing TIPS [3]. The majority of these models have been applied to heterogeneous groups of patients undergoing TIPS procedures with different indications, including variceal bleeding or refractory ascites. Commonly used prognostic models include the Child-Pugh score; the creatinine-modified Child–Pugh score (or Child–Creatinine score) [12]; the model for end-stage liver disease (MELD); the revised version of the MELD score (the “MELD-Na score”) [13], [14]; the acute physiology and chronic health evaluation (APACHE II) score [15]; the Bonn TIPSS early mortality (BOTEM) score [16]; the Emory score [17], and others. Several factors associated with poor survival have been identified, including hyperbilirubinemia, hyponatremia, platelet count, and episodes of hepatic encephalopathy without any triggering events [18], [19]. Most models were developed in patients receiving bare stents; few studies have focused on patients with covered stents. Most of the models are complicated and rarely used outside of a clinical trial setting. Uncertainty concerning the relative importance of the covered stents remains, and an evidenced-based improvement may be warranted in the era of covered stents.

The aim of this study was to establish a clinically usable predictive model for one-year survival in patients undergoing TIPS procedures with the use of covered stents. Using a Cox proportional regression hazards model, we developed a novel score to predict patient outcome using characteristics of the patients derived prior to their TIPS procedure. This new model was then validated in an independent cohort of patients with covered stents.

## Patients and Methods

### Ethics Statement

The study protocol conformed to the ethical guidelines of the 1975 Declaration of Helsinki and was approved by the ethics committee of Xijing Hospital. Written informed consent was obtained from each patient.

### Patients

While this study is retrospective, the data was collected in a prospective manner. A total of 124 cirrhotic patients (80 men and 44 women; median age, 46 years; range, 21–88 years) with symptomatic portal hypertension (SPH) were referred to our unit. TIPS procedures were performed with PTFE-covered stents in these patients from July 2010 to December 2011; these patients were designated the training cohort (TC).

The exclusion criteria were as follows: age younger than 18 years, hepatocellular carcinoma or other cancers, the previous use of a portosystemic shunt or TIPS, portal vein thrombosis, and Budd–Chiari syndrome.

The results of the analysis in the TC were assessed in an independent validation cohort (VC). This cohort included 86 patients with cirrhosis and SPH who were treated with covered stents from January 2012 to June 2012 (to have a follow-up period longer than one year). The selection criteria for the validation cohorts were the same as for the training cohort.

### TIPS Procedures

All of the procedures were performed in a dedicated sterile interventional radiology suite by the same team; conscious sedation and local anesthesia was using during the procedure. All TIPS used a standard right transjugular appraoch to coordinate with indirect portography via the superior mesenteric artery. A 10F RUPS-100 (Cook, Bloomington, IN, USA) was used in all TIPS procedures to gain access to the portal vein, and the shunt was mostly created between the right hepatic vein and the left branch of the portal vein. Preoperative and postoperative portosystemic pressure gradients (PSG) were measured. Fluency covered stents (Bard / Angiomed GmbH & Co, Karlsruhe, Germany) were used. The intention of the operators was to extend the cephalic end of the stent to the hepatocaval junction and make the caudal end of the stent parallel to the vascular wall of the portal vein.

After the TIPS procedure was performed, intravenous heparin (8000–12,000 U/d) was administered for five to seven days, warfarin was administered for the following six months, and aspirin was prescribed for life. The dosages were prescribed to achieve an international normalized ratio (INR) of up to two times the upper limit of normal to prevent shunt dysfunction[20], [21].

### Definitions

Cirrhosis was diagnosed based on a history of liver disease, clinical presentation, laboratory testing, and imaging studies. Confirmation was obtained with a liver biopsy if a diagnosis of cirrhosis was inconclusive [22], [23].

The MELD-Na score was calculated with the following formula [14]: MELD-Na  =  MELD − Na − [0.025× MELD ×(140 − Na)] +140, where the serum Na is bounded between 125 and 140 mmol/L [14].

The Child–Creatinine score was derived from the original Child–Pugh score by adding one point for creatinine levels ≤1.1 mg/dL (97.24 µmol/L), two points for creatinine levels of 1.2–1.8 mg/dL (106.08-159.12 µmol/L), and three points for creatinine levels >1.8 mg/dL (159.12 µmol/L) [12].

The serum sodium (Na) concentration was classified as normonatremic (serum [Na^+^] >135 mmol/L), mildly hyponatremic (serum [Na^+^] 130–135 mmol/L) and severely hyponatremic (serum [Na^+^] <130 mmol/L) [24].

Ascites was classified into grades of one to three based on the definitions of the International Ascites Club [25]. Refractory ascites was defined as being unresponsive to a sodium-restricted diet and intensive diuretic treatment, intolerant of diuretic therapy, and rapidly recurring after therapeutic paracentesis [25].

### Follow-up

All of the subjects were evaluated and followed by the same medical team using a prospective protocol and surveillance strategy. Follow-up visits, which involved the administration of routine blood tests, coagulation function tests, liver and renal function tests, measurement of electrolytes, upper gastrointestinal endoscopy, and color Doppler ultrasound (diameter, flow velocity, and direction of flow in the portal vein and shunt), were scheduled one, three, and six months after the TIPS procedure and every six months thereafter. The patients were followed from the date of their TIPS procedure until death, liver transplantation, or study closure (May 31, 2013). Patients were considered lost to follow-up if they did not come to two consecutive six-month clinical examinations.

### Statistics

The continuous variables are presented as medians and ranges and were compared using Student’s t-test. The categorical data are expressed as absolutes and percentage values and were compared using the chi-squared test. The patients who underwent transplantation were censored at the corresponding time. The prognostic models were generated using one-year mortality as the primary end point (i.e., follow-up time was limited to 12 months). The cumulative survivals were determined with the Kaplan-Meier method and compared using the log-rank test. A Cox proportional regression hazards model was used to assess the prognostic value of the baseline variables in the univariate and multivariate analysis. To compare the ability of different scores to accurately predict one-year survival following TIPS, the concordance c-statistic (area under the receiver operating characteristic curve (ROC)) was calculated. This statistic ranges from 0 to 1, and scores of >0.7 are generally indicative of a useful predictive model. P-values of less than 0.05 were considered to indicate statistical significance, and all of the tests were two-sided. The statistical software package SPSS 16.0 (SPSS, Chicago, Illinois, USA) was used for the analysis.

## Results

The patient characteristics of the two cohorts are shown in Table 1. No patient was lost to follow-up in either the TC or the VC. No patients in the TC or VC underwent transplantation within one year. The two cohorts were very similar at baseline for all factors except INR and creatinine (Table 1).

**Table 1:** Demographic, clinical, and biochemical characteristics of cirrhotic patients with SPH who underwent TIPS with covered stents.

Variables	TC (n = 124)	VC (n = 86)	P value
Age, years	46 (21–88)	49 (27–77)	0.082
Gender, male/female	80/44	66/20	0.066
Etiology			0.55
HBV	99 (79.8%)	66 (76.7%)	
Alcohol	6 (4.8%)	4 (4.7%)	
Others	12 (9.7%)	9 (10.5%)	
Unknown	7 (5.7%)	7 (8.1%)	
Child-Pugh score	7 (5–12)	7 (5–12)	0.327
Child-Pugh class, A/B/C	45/54/25	32/46/8	0.098
Child-Creatinine score	9 (6–14)	8 (6–13)	0.359
MELD score	9.35 (0.53–26.8)	10.75 (2.03–21.81)	0.244
MELD-Na score	11.16 (0.42–28.01)	11.8 (1.3–27.38)	0.981
INR	1.43 (0.98–7.48)	1.34 (0.97–2.93)	**0.032**
Serum albumin, g/L	33.5 (17.3–44.4)	32.8 (21.2–48.1)	0.601
Serum bilirubin, µmol/L	22.25 (4.90–68.4)	20.9 (5.3–105.9)	0.536
Serum creatinine, µmol/L	72 (32–282)	88.5 (41–281)	**0.001**
Serum sodium, mmol/L	139.05 (118.1–146.3)	138.7 (126.4–147.5)	0.604
Leukocytes, x10^9^/L	3.31 (0.71–18.6)	2.66 (0.36–15.04)	0.054
Hemoglobin, g/L	86 (20–134)	80.5 (47–822)	0.25
Platelets, x10^9^/L	56.5 (2–284)	58 (18–318)	0.69
Indication for TIPS			0.067
Variceal bleeding	107	81	
Refractory ascites	17	5	
Ascites, absent/1/2/3	39/45/16/24	25/40/10/11	0.571

**NOTE**. Values are expressed as the median (range) or frequency (percentage). Bold values indicate P-values <0.05. **Abbreviations**: HBV, hepatitis B virus; HCV, hepatitis C virus; INR, international normalized ratio; SPH, symptomatic portal hypertension; TC, training cohort; TIPS, transjugular intrahepatic portosystemic shunt; VC, validation cohort.

### Survival

The causes of death for the two cohorts are presented in Table 2. There were 25 (20.2%) and 13 (15.1%) deaths within one year in the TC and VC, respectively, and the cumulative survival rate was 80.6% and 84.9%, respectively. Causes of mortality were similar for the TC and VC (p = 0.372).

**Table 2:** Causes of death within one year in the training cohort (n = 25) and validation cohort (n = 13).

Cause of death	TC (%)	VC (%)
Liver failure	7 (28%)	3 (23%)
Variceal bleeding	6 (24%)	2 (15%)
Hepatic encephalopathy	3 (12%)	2 (15%)
Multiorgan failure	4 (16%)	1 (8%)
Hepatorenal syndrome	3 (12%)	3 (23%)
Hemoperitoneum	1 (4%)	0 (0%)
Others	1 (4%)	2 (15%)

**Note**. Values are expressed as frequencies (percentage). **Abbreviations**: TC, training cohort; VC, validation cohort.

A series of clinical and biochemical parameters were analyzed to identify variables that were significantly associated with one-year survival. The results of the univariate and multivariate analyses are presented in Table 3. We included the Child-Pugh and MELD scores in place of INR, albumin, bilirubin, creatinine values, and ascites characteristics (which were the components of the Child-Pugh and MELD scores in the multivariate analysis for one-year survival) to avoid the strong correlation that exists between these variables. As a result, the Child-Pugh score and serum Na levels were significantly associated with one-year survival.

**Table 3:** Univariate and multivariate analyses of pre-TIPS prognostic factors associated with one-year survival in patients in the training cohort with covered stents.

Variables	Univariate analysis			Multivariate analysis		
	HR	95% CI	P-value	HR	95% CI	P-value
Age	1.038	1.009–1.067	**0.010**	1.025	0.992–1.060	0.132
Sex	2.011	0.903–4.477	0.087	—	—	—
Ascites (yes/no)	0.062	0.014–0.273	**0.000**	—	—	—
Hemoglobin	1.005	0.987–1.023	0.577	—	—	—
Platelet count	1.001	0.992–1.010	0.758	—	—	—
INR	1.360	1.009–1.833	**0.043**	—	—	—
Serum albumin	0.837	0.774–0.904	**0.000**	—	—	—
Total Bilirubin	1.033	1.008–1.060	**0.011**	—	—	—
ALP	1.005	1.001–1.009	**0.014**	1.006	1.000-1.011	0.054
Serum creatinine	1.015	1.005–1.024	**0.003**	—	—	—
Serum potassium	1.158	0.430–3.119	0.771	—	—	—
Serum sodium	3.081	1.347–7.044	**0.008**	3.243	1.338–7.861	**0.009**
Child-Pugh score	1.540	1.248–1.901	**0.000**	1.501	1.103–2.044	**0.010**
MELD score	1.123	1.044–1.207	**0.002**	1.044	0.943–1.157	0.406

**NOTE**. Bold values indicate P-values <0.05; —: not included in the multivariate analysis. **Abbreviations**: INR, international normalized ratio; ALP, alkaline phosphatase.

### Child-Na Score: Model Development

Incorporating the serum Na into the Child-Pugh score may improve the accuracy of the Child-Pugh score. We developed a new score, the “Child-Na score”, to incorporate the effect of serum Na (as a categorical variable) while keeping the Child-Pugh score unchanged; the Child-Pugh score is calculated by combining these two prognostic values with the corresponding regression coefficients (B-values, 0.406 for Child-Pugh score and 1.176 for Na). The B-values were multiplied by 2.5 and rounded to facilitate the calculation of the Child-Na score using the following formula: Child-Na Score  =  Child-Pugh score +3× (serum Na classification: 1 in cases of normonatremia, 2 in cases of mild hyponatremia, 3 in cases of severe hyponatremia).

### Comparison of the Child-Na, Child-Pugh, Child-Creatinine, MELD, and MELD-Na scores

**Figure 1:**
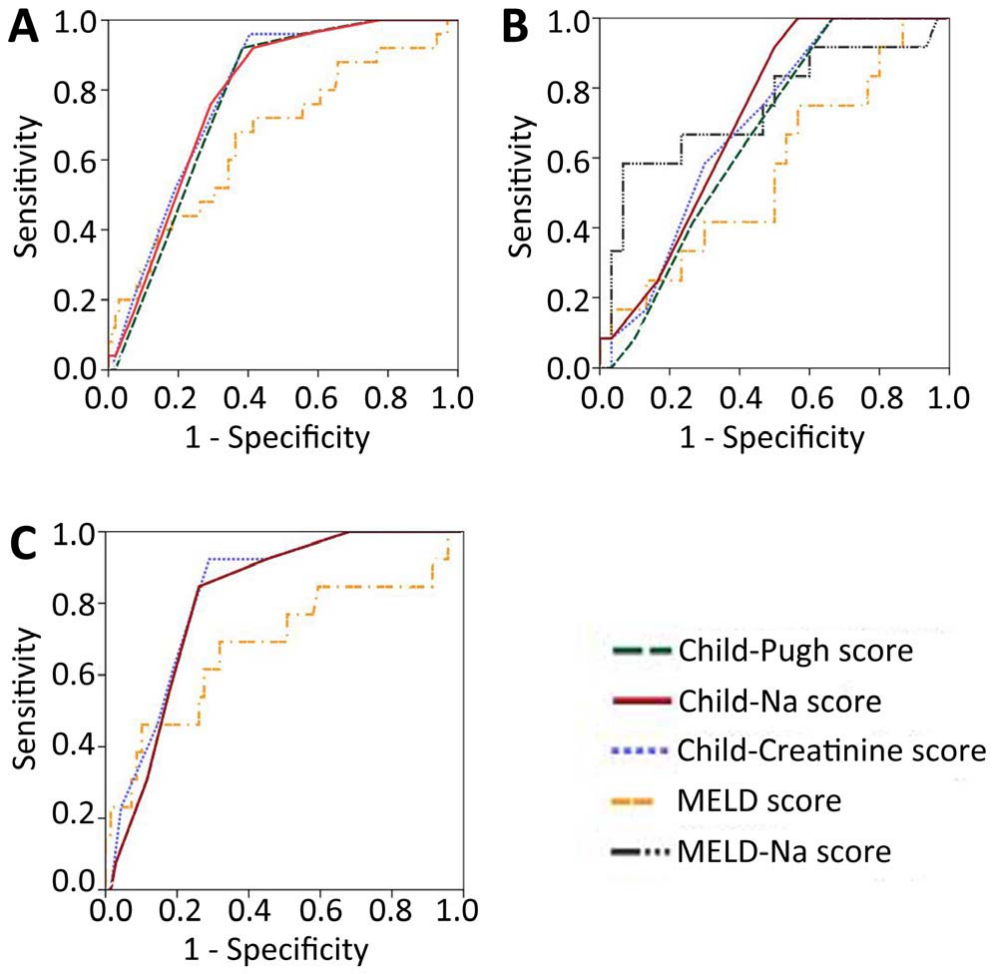
Comparison of the predictive values of the Child-Na, Child-Creatinine, Child-Pugh, MELD, and MELD-Na scores for one-year survival for all patients (A), for patients with serum Na levels <138 mmol/L (B), and for patients with serum Na levels ≥138 mmol/L (C). **Note:** The MELD-Na score was not calculated in A and C because this model requires a serum Na concentration between 125 and 140 mmol/L.

The c-statistics for the Child-Na, Child-Pugh, Child-Creatinine, and MELD scores for one-year survival in the entire sample of patients are shown in Table 4 (the MELD-Na score was not calculated because this score required a serum Na concentration between 125 and 140 mmol/L). The c-statistics for each score were ranked as follows: Child-Creatinine > Child-Na > Child-Pugh > MELD (Table 4, Fig. 1A). The former three scores have values greater than 0.7 and were therefore considered indicative of useful predictive models; however, the difference between these values was very small.

**Table 4:** ROC curve analysis of the Child-Na score, Child-Pugh score, Child-Creatinine score, MELD score, and MELD-NA score, according to the level of serum Na.

	All patients		Serum Na <138 mmol/L		Serum Na ≥138 mmol/L	
	c statistic	P	c statistic	P	c statistic	P
Child-Na	0.784	<0.001	0.76	0.009	0.806	<0.001
Child-Pugh	0.764	<0.001	**0.663**	0.103	0.806	<0.001
Child-Creatinine	0.785	<0.001	**0.694**	0.051	0.824	<0.001
MELD	**0.654**	0.018	**0.564**	0.522	**0.685**	0.035
MELD-NA	―	―	0.746	0.014	―	―

**Note**.―, MELD-Na was developed when the serum sodium concentration ranged from 125 to 140 mmol/L.

The predictive accuracy of the Child-Na and MELD-Na scores varied when several upper limits for serum Na were considered. When the upper limits for serum Na were set at 138 mmol/L, the c-statistics for each score were ranked as follows: Child-Na > MELD-Na > Child-Creatinine > Child-Pugh > MELD (Table 4, Fig. 1B). Only the Child-Na and MELD-Na scores had a c-statistic >0.7 and a P-value <0.05, and the difference between the c-statistics of these models was also very small (c-statistic: 0.014) (Table 4, Fig. 1B). For patients with serum Na ≥138 mmol/L, the c-statistics for each score were ranked as follows: Child-Creatinine > Child-Na  =  Child-Pugh > MELD (Table 4, Fig. 1C).

### Cut-off Value of Child-Na Score in Patients with Na Levels <138 mmol/L

The sensitivity, specificity, positive likelihood ratio, negative likelihood ratio, positive predictive value, and negative predictive value of the Child-Na score for different thresholds are listed in table 5. We selected a cut-off value of 15.5 points.

**Table 5:** Criterion values and coordinates of the ROC curve for the Child-Na score to predict one-year survival for different cut-off values observed in the training cohort.

Cut-off	Sensitivity	Specificity	+LR	-LR	+PV	-PV
13.5	53.33	75.00	2.13	0.62	84.2	39.1
14.5	63.33	66.67	1.90	0.55	82.6	42.1
15.5	83.33	50.00	1.67	0.33	80.6	54.5
16.5	90.00	41.67	1.54	0.24	79.4	62.5

**Note**. +LR, positive likelihood ratio; -LR, negative likelihood ratio; +PV, positive predictive value; -PV, negative predictive value.

### The Child-Na Score Predicts One-year Survival in the Training and Validation Cohorts

**Figure 2:**
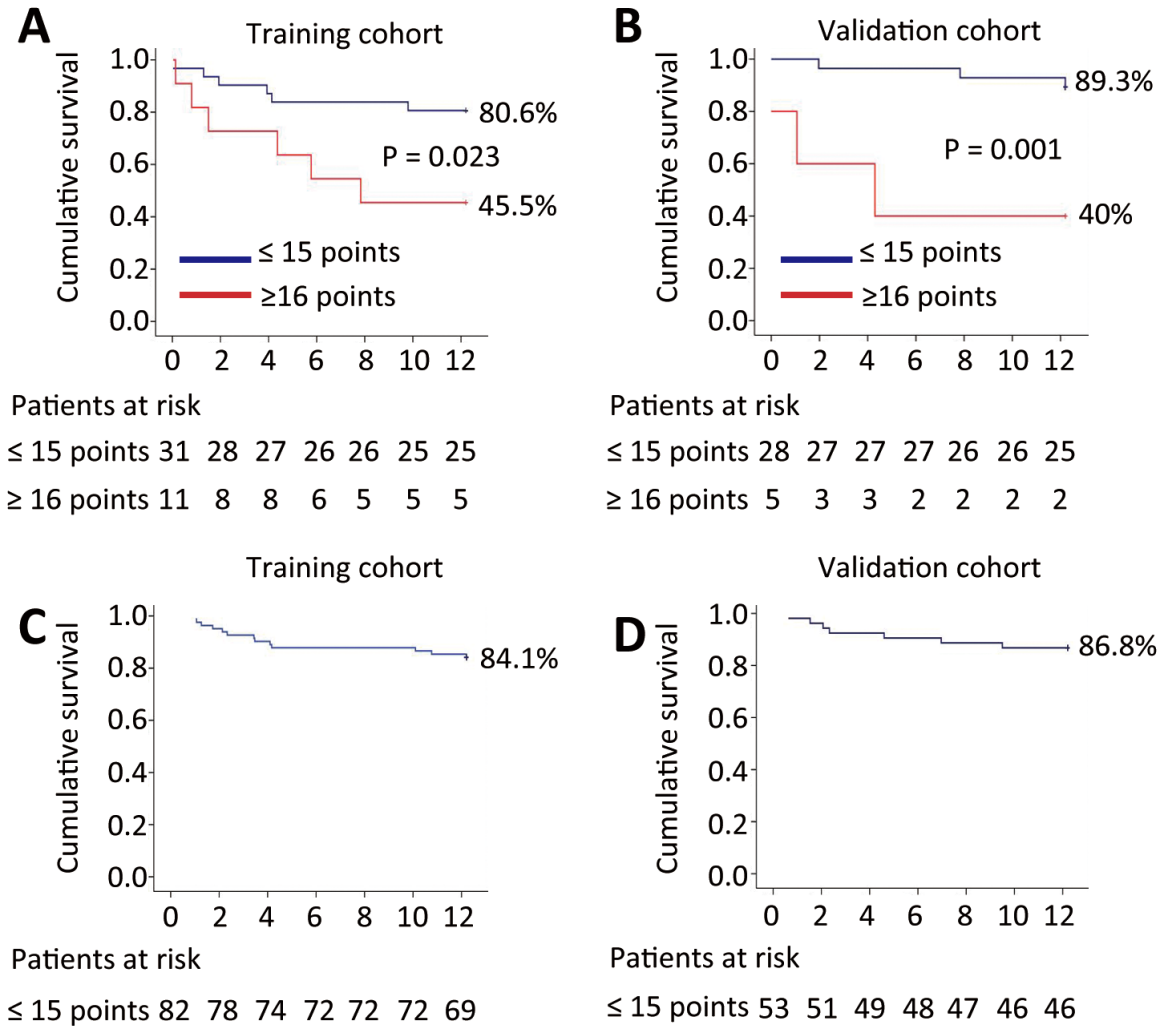
The one-year cumulative survival following the TIPS procedure of patients in the two Child-Na score groups (≤15 points, ≥16 points) in the training cohort (A and C) and in the validation cohort (B and D). **Note:** All of the patients with serum Na levels ≥138 mmol/L had a Child-Na score ≤15 points.

In the TC, the Child-Na score identified two subgroups of patients with distinct prognoses in patients with serum Na levels <138 mmol/L. The actual one-year survival rate in patients with a Child-Na score ≤15 points (n = 31) was 80.6%, while patients with a Child-Na score ≥16 (n = 11) had survival rates of 45.5% (log rank-test: p = 0.023) (Fig. 2A). Similar results were found in the independent VC. The cumulative one-year survival rate of patients with a Child-Na score ≤15 points (n = 28) was 89.3%, and the one-year survival rate for patients with a Child-Na score ≥16 (n = 5) was 40% (log rank-test: p = 0.001) (Fig. 2B). Patients with serum Na levels ≥138 mmol/L all had Child-Na scores below 15 points, and the cumulative one-year survival rates for these patients were 84.1% and 86.8% in the TC and VC, respectively (Fig. 2C and D).

### Subgroup analysis

**Figure 3:**
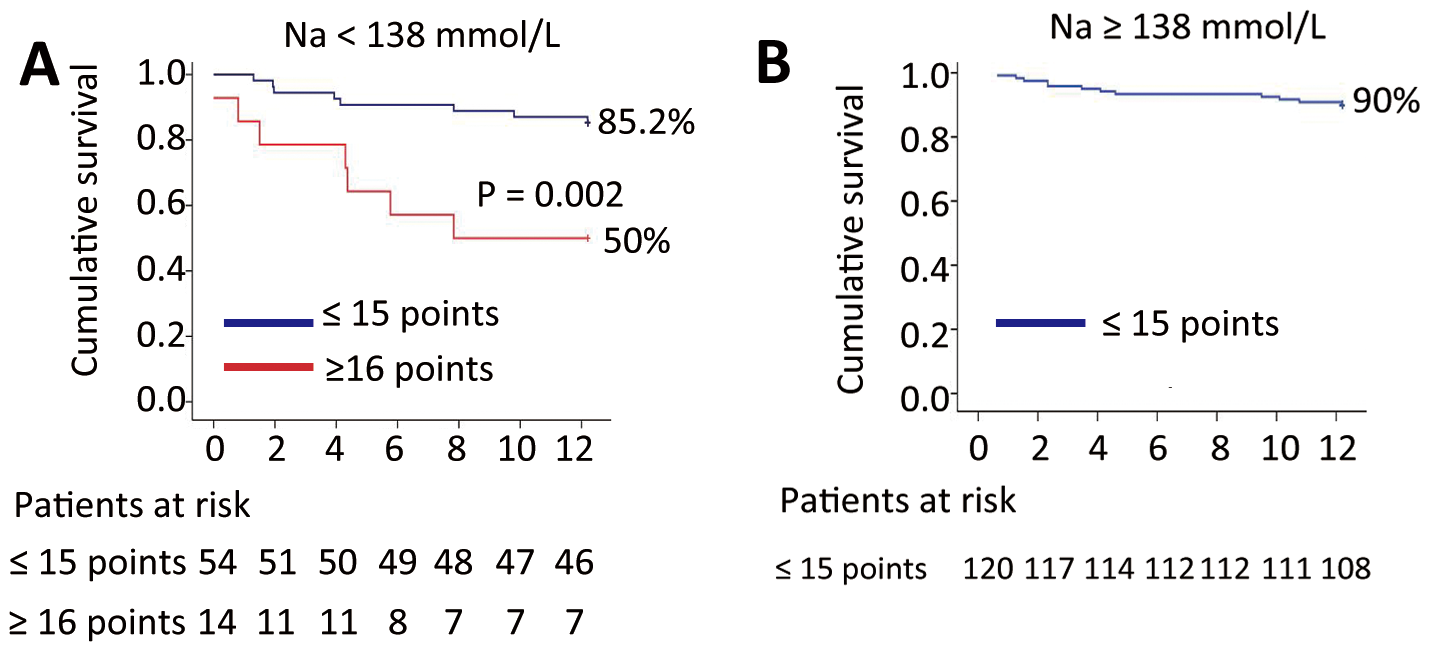
Subgroup analysis of one-year cumulative survival in patients pooled from the training cohort and validation cohort undergoing TIPS with variceal bleeding.

All of the patients with serum Na levels <138 mmol/L were pooled from the TC and VC and stratified by indication for TIPS, i.e., variceal bleeding or refractory ascites; the results of this analysis are shown in Figure 3. The cumulative survival rates of the subgroup with variceal bleeding and Child-Na scores of ≤15 (n = 54) and ≥16 (n = 14) was 85.2% and 50% (p = 0.002), respectively (Fig. 3A). In the subgroup of patients with refractory ascites, all of the patients with Child-Na scores ≥16 died (2/2) within one year, while only one patient with a Child-Na score ≤15 died (1/5). The Child-Na scores of all of the patients with serum Na levels ≥138 mmol/L were ≤15, and the cumulative one-year survival rate was 90% for patients with variceal bleeding and and 46.7%(7/15) for patients with refractory ascites (p = 0.002) (Fig. 3 B).

## Discussion

Over the last two decades, several scoring systems for predicting survival in patients with cirrhosis and SPH following TIPS have been developed; however, few of these models have gained widespread acceptance [12]–[19]. Most of these models were developed in patients with bare stents; few studies of patients treated with covered stents have been performed [4], [11], [26]. Furthermore, many of these scores are cumbersome and rarely used outside of a clinical trial setting. The identification of patients at high risk of death after a TIPS procedure with covered stents is still being investigated. We developed a new model, the “Child-Na” score, the performance of which was superior to that of other models in patients with serum Na levels <138 mmol/L. The validity of this model was successfully confirmed in an independent validation cohort.

The Child-Pugh score and the MELD score are the most commonly used prognostic models in patients with cirrhosis. Attempts have been made to improve these models by either adding new variables (such as serum creatinine and Na) or by using more sophisticated measures [13], [27]–[30]. A number of investigations have documented the importance of serum Na as an independent predictor of death (23, 27) which is reliable, quantitative, reproducible, and objective, and represents reasonable candidates for inclusion in prognostic models (27). The traditional Child-Pugh score could be improved by adding serum creatinine, but this score has not been proven to be more accurate than the MELD score [12]. The MELD score was first developed to predict three-month mortality in patients undergoing TIPS procedures [31]. Recently, an MELD-based model, the “MELD-Na score”, was used to determine liver allocation [13], [14], [30].

In the present study, we compared the prognostic accuracy of the Child-Na score with that of the Child-Pugh, Child–Creatinine, MELD, and MELD-Na scores. When all patients were included in the analysis, we observed that the Child-Pugh based scores were more accurate than other scores. Furthermore, we compared the five scores in two subgroups stratified by serum Na levels (threshold of 138 mmol/L). Similar results were obtained in patients with serum Na levels ≥138 mmol/L. In the subgroup of patients with serum Na levels <138 mmol/L, only the Child-Na and MELD-Na models had similar c-statistics of >0.7. Recently, Gaba et al. also compared prognostic capability of different liver disease scoring systems for prediction of early mortality after TIPS creation. However, they concluded that MELD and MELD-Na scores most effectively predict survival after TIPS creation [26]. However, the MELD-Na score cannot be calculated at the bedside, nor can it be applied in everyday clinical practice. Thus, the Child-Na score should be further evaluated, as it is simpler and more clinically practical than some of the other scores.

In patients with serum Na levels <138 mmol/L, the Child-Na score was able to identify two groups of patients with distinctly different prognoses. Patients with scores of 15 points or fewer before the TIPS procedure had a significantly higher one-year survival rate than patients with scores ≥16 points. Furthermore, patients with variceal bleeding and a Child-Na score ≤15 prior to the TIPS procedure had a better prognosis than the other patients. Patients presenting with refractory ascites and a Child-Na score ≥16 may be not suitable candidates for TIPS.

### Limitations

This study had some limitations, primarily related to its retrospective nature. The data on the patients who underwent TIPS procedures were rigorously collected following a prospective protocol for a diagnostic work-up and a surveillance strategy at the time of TIPS insertion and during follow-up. Serum Na concentration can depend on a number of factors, including fluid overload and the use of diuretics. Published data and common clinical observations indicate that hyponatremia in patients with cirrhosis is difficult to alter [32], [33]. The relatively small sample size in this subgroup of patients is another limitation.

### Conclusion

We developed and validated a novel, simple, noninvasive, clinically applicable prognostic score, the Child-Na score, for patients with cirrhosis and SPH undergoing TIPS procedures. Patients with variceal bleeding and a Child-Na score ≤15 had a better prognosis, regardless of serum Na levels, than patients with a score ≥16. Patients with refractory ascites and a Child-Na score ≥16 had a very high risk of death after the TIPS procedures; these patients should be treated with caution. Patients with even higher scores should receive other evidence-based treatments to avoid an ineffective and expensive procedure. Further studies with a larger sample size are warranted to test this new model, especially in a prospective clinical trial.
